# Vagus nerve stimulation in children with drug-resistant epilepsy of monogenic etiology

**DOI:** 10.3389/fneur.2022.951850

**Published:** 2022-09-01

**Authors:** Han Xie, Jiayi Ma, Taoyun Ji, Qingzhu Liu, Lixin Cai, Ye Wu

**Affiliations:** ^1^Department of Pediatrics, Peking University First Hospital, Beijing, China; ^2^Pediatric Epilepsy Center, Peking University First Hospital, Beijing, China

**Keywords:** vagus nerve stimulation, children, monogenic etiology, drug resistant epilepsy, efficacy

## Abstract

Vagus nerve stimulation (VNS) is an effective treatment for drug-resistant epilepsy (DRE). The present study evaluated the efficacy of VNS in pediatric patients with DRE of monogenic etiology. A total of 20 patients who received VNS treatment at our center were followed up every 3 months through outpatient visits or a remote programming platform. The median follow-up time was 1.4 years (range: 1.0–2.9). The rate of response to VNS at 12 months of follow-up was 55.0% (11/20) and the seizure-free rate was 10.0% (2/20). We found that 75.0% (3/4) of patients with an *SCN1A* variant had a >50% reduction in seizure frequency. Patients with pathogenic mutations in the *SLC35A2, CIC, DNM1, MBD5, TUBGCP6, EEF1A2*, and *CHD2* genes or duplication of X q28 (*MECP2* gene) had a >50% reduction in seizure frequency. Compared with the preoperative electroencephalography (EEG), at 6, 12, 18, and 24 months after stimulator implantation, the percentage of the patients whose background frequency increased >1.5 Hz was respectively, 15.0% (3/20), 50.0% (10/20), 58.3% (7/12) and 62.5% (5/8); the percentage of the patients whose interictal EEG showed a >50% decrease in spike number was respectively 10% (2/20), 40.0% (8/20), 41.6% (5/12) and 50.0% (4/8). In the 9 patients with no response to VNS treatment, there was no difference in terms of spike number and background frequency between preoperative and postoperative EEG. Five of the 20 children (25.0%) reached new developmental milestones or acquired new skills after VNS compared to the preoperative evaluation. The efficacy of VNS in pediatric patients with DRE of monogenic etiology is consistent with that in the overall population of pediatric DRE patients. Patients with Dravet syndrome (DS), tuberous sclerosis complex (TSC), or Rett syndrome/*MECP2* duplication syndrome may have a satisfactory response to VNS, but it is unclear whether patients with rare variants of epilepsy-related genes can benefit from the treatment.

## Introduction

Anti-seizure medications (ASMs) are the first-line treatment for controlling epileptic seizures, but 30% of patients with epilepsy are drug-resistant (1, 2). In patients with drug-resistant epilepsy (DRE) who fail to respond to etiologic treatments (e.g., lesion resection for structural etiologies, immunotherapy for autoimmune etiologies), a ketogenic diet (KD) or neuromodulation (e.g., vagus nerve stimulation [VNS]) is considered as an alternative therapy. VNS modulates the neural network by electrically stimulating the vagus nerve; it was approved for the treatment of DRE by the European Medicines Agency in 1994 and by the China Food and Drug Administration in 2008.

Monogenic etiologies of DRE include pathogenic variants of genes encoding ion channels, receptors, and synaptic proteins (3). Previous studies have mostly focused on the efficacy of VNS in patients with DRE harboring variants of the *SCN1A* or *TSC1*/*TSC2* gene, but there have been few studies in patients with DRE of other monogenic etiologies. The aim of the present cohort study is to analyze the efficacy and clinical outcomes of VNS in children with DRE of any monogenic etiologies.

## Methods

### Patients

The study enrolled 20 children with DRE of monogenic etiology who underwent VNS (102, LivaNova; G111 or G112, PINS Medical, Beijing, China) at the Pediatric Epilepsy Center of Peking University First Hospital from January 2018 to December 2020. The inclusion criteria were as follows: (1) age <18 years at the time of implantation; (2) monogenic DRE confirmed by whole-exome sequencing, or epilepsy due to chromosome copy number variation (CNV) confirmed by array comparative genomic hybridization or CNV sequencing; and (3) follow-up period of at least 1 year after implantation. Patients were excluded if they underwent epilepsy surgery (e.g., corpus callotomy) or KD or glucocorticoid therapy during VNS therapy. The Medical Ethics Committee of Peking University First Hospital approved this study. The parents of each patient signed the informed consent form.

### Clinical data collection and follow-up

Our study is an ambispective cohort study. Retrospective data were collected from the hospital records, and all enrolled children were prospectively followed up for more than 1 year. Preoperative (baseline) data were collected for each patient including sex, age at seizure onset, seizure course before implantation, seizure type and frequency, epileptic syndrome, developmental assessment, ASMs, gene variants, and electroencephalography (EEG). Patient follow-up was done through the outpatient department of Peking University First Hospital or a remote programming platform. The last follow-up visit was in January 2022. Postoperative (follow-up) data included seizure frequency, developmental assessment, ASMs, EEG and VNS parameters.

### Definition of VNS response and seizure freedom

A responder was defined as a patient with a >50% reduction in seizure frequency after VNS compared with the preoperative baseline value. The responder rate was defined as the percentage of the responders. Seizure freedom was defined as freedom from seizures for at least 6 months. The preoperative (baseline) seizure frequency was calculated as the mean number of seizures over the 3-month period before implantation, and the seizure frequency at each follow-up visit was the mean number of seizures over the 3-month period preceding the visit.

To analyze the relationship between seizure types and VNS efficacy in these patients, we calculated the VNS efficacy of each seizure type separately. Response rate of each seizure type was defined as the percentage of patients with a >50% reduction in corresponding seizure after VNS compared with the preoperative baseline value.

### VNS programming procedure

VNS parameters were adjusted during outpatient visits or through the VNS remote programing platform (4, 5). The VNS remote programming platform included a real-time video chat between neurologists and the family of the patient, which was convenient for neurologists to obtain clinical data of children, such as the frequency of seizures and medication. We regularly arranged for outpatient visits every 6 months after VNS implantation to do 4-h video EEG and developmental assessment. For each VNS-treated patient, the stimulator was turned on about 14 days after implantation; the simulation parameters in conventional mode at startup were as follows: amplitude = 0.5 mA, pulse width = 250 or 500 μs, frequency = 30 Hz, stimulation time =30 s, and interval time = 5 min. The pulse amplitude was increased by 0.2–0.5 mA every 2 weeks and gradually adjusted to 1.5 mA. Thereafter, pulse amplitude continued to be increased or the duty cycle was gradually increased.

### Spike counting and analysis of background frequency

During the follow-up, all patients had 4-h video EEG every 6 months. Each EEG evaluation of the patients in this study was evaluated by the epileptologists. The epileptologists counted spike number on interictal EEG and analyzed background frequency. Compared with preoperative EEG, if the patients had a >50% reduction of spike number or the background frequency of the patients increased >1.5 Hz, we would consider that the EEG of these patients were improved.

### Statistical analysis

Continuous variables were non-normally distributed according to the Shapiro–Wilk test, and are described as median value and range. Descriptive statistics were used to summarize patient characteristics. Categorical variables were summarized as numbers and as percentages of the total number of patients in each category. The χ^2^ test and Fisher's exact test were used to evaluate the goodness of fit between the theoretical and actual frequency distributions. The non-parametric Mann–Whitney U-test (two categories) was used for inter-group comparisons of non-normally distributed variables. Differences with a *P*-value <0.05 were considered statistically significant. All statistical tests were performed using SPSS Statistics (IBM, Armonk, NY, USA).

## Results

### Baseline data of patients before stimulator implantation

From January 2018 to December 2020, 138 children with DRE underwent stimulator implantation at our pediatric epilepsy center. A total of 117 patients who received VNS treatment were regularly followed up for >1 year. Of these patients, 23 were diagnosed with monogenic epilepsy, and 20 (10 male and 10 female) met the inclusion criteria for this study. The median age of seizure onset was 5.5 months (range: 0.3–105 months). The median age at stimulator implantation was 55 months (range: 19–201 months). The median seizure course before implantation was 40 months (range: 11–104 months). Pathogenic genes were *SCN1A* (*n* = 4), *DNM1* (*n* = 2), *SLC35A2* (*n* = 1), *CIC* (*n* = 1), *MBD5* (*n* = 1), *TUBGCP6* (*n* = 1), *EEF1A2* (*n* = 1), *STXBP1* (*n* = 1), *LGI1* (*n* = 1), *GRIN2D* (*n* = 1), *RANBP2* (*n* = 1), *TSC2* (*n* = 1), *SZT2* (*n* = 1), *CHD2* (*n* = 1), *CHRNA4* (*n* = 1), and *MECP2* (*n* = 1). The most common epilepsy syndromes were Lennox–Gastaut syndrome (6/20, 30%) and Dravet syndrome (DS) (4/20, 20%). The median number of ASMs ever tried before stimulator implantation including adrenocorticotropic hormone and KD was 6 (range: 3–10). For development assessment, 75% (15/20) had severe development delay (DD)/intellectual disability (ID), 10% (2/20) had moderate DD/ID, 15% of patients (3/20) had mild DD/ID, and preoperative data for the patients were shown in Table 1.

**Table 1:** Clinical and genetic information of the 20 children with monogenic etiologies of DRE.

**PN**	**Gender**	**Age at seizure onset**	**Age at VNS implantation**	**Seizure type /epilepsy syndrome**	**Development at VNS implantation**	**ASM /other treatments before VNS**	**Pathogenic gene; inheritance**	**Phenotype (MIM number)**	**Seizure outcome at last visit**
1	M	4 m	3 y 6 m	GTCS, F/DS	Severe DD	VPA, LTG, LEV, TPM, PER	*SCN1A*; AD	Dravet syndrome (607208)	60% reduction in seizures
2	F	6 m	6 y 1 m	GTCS, F /DS	Severe DD	VPA, TPM, LEV, OXC, ZNS, CLB	*SCN1A;* AD	Dravet syndrome (607208)	60% reduction of seizures
3	F	6 m	4 y 9 m	F, M/DS	Severe DD	VPA, OXC, LEV, LTG, TPM, CLB, CZP	*SCN1A*; AD	Dravet syndrome (607208)	60% reduction in seizures
4	F	3 m	7 y 11 m	F, GTCS/DS	Severe ID	LEV, OXC, PB, LTG, TPM, ZNS	*SCN1A*; AD	Dravet syndrome (607208)	No reduction in seizures
5	F	15 d	1 y 7 m	F, S	Severe DD	LTG, TPM, VGB, VPA	*DNM1*; *AD*	Developmental and epileptic encephalopathy, type 31 (616346)	60% reduction in Seizures
6	F	14 d	2 y 3 m	F, S	Severe DD	CBZ, LTG, TPM, VGB, CZP, PB, VPA, LEV	*DNM1*; *AD*	Developmental and epileptic encephalopathy, type 31 (616346)	No reduction in seizures
7	M	8 y 6 m	9 y 5 m	M, A, AA, T/LGS	Severe DD	VPA, LTG, LEV, PER, CLB	0.331Mb duplication of X q28 (*MECP2*); *XLD*	Rett syndrome (312750)	Seizure freedom
8	M	1 y 1 m	4 y 7 m	S, F, AA	Severe DD	VPA, TPM, CZP, KD, RFM, CLB, LCM	*SLC35A2; XLD*	Developmental and epileptic encephalopathy, type22 (300896)	Seizure freedom
9	M	3 y 2 m	4 y 10 m	T, M, AA/LGS	Severe DD	VPA, LTG, LEV	*CIC;* AD	Mental retardation, autosomal dominant 45 (617600)	75% reduction of seizures
10	F	3 y 4 m	4 y 4 m	F, GTCS	Moderate DD	LEV, VPA, CZP, TPM	*MBD5;* AD	Intellectual developmental disorder, autosomal dominant 1 (156200)	90% reduction in seizures
11	M	7 m	5 y 3 m	F, AA	Mild ID	LEV, VPA, LTG, TPM, PER, KD	*TUBGCP6;* AR	Microcephaly and chorioretinopathy, autosomal recessive, 1 (251270)	60% reduction in seizures
12	M	4 m	3 y 1 m	M, A	Mild DD	VPA, LTG, LEV, CZP, TPM	*EEF1A2*; *AD*	Developmental and epileptic encephalopathy 33 (616409)	80% reduction in seizures
13	F	5 m	9 y 1 m	AA, M, T, A, S	Moderate ID	VPA, LEV, CZP, TPM, LTG, VGB, RFM	*CHD2;* AD	Developmental and epileptic encephalopathy 94 (615369)	90% reduction in seizures
14	F	8 y 9 m	16 y 9 m	F	Mild ID	VPA, LTG, LCM, CBZ, OXC, TPM, LEV	*LGI1;* AD	Epilepsy, familial temporal lobe, 1 (600512)	No reduction in seizures
15	M	3 m	4 y 7 m	T, S	Severe DD	VPA, TPM, CZP, VGB, LTG, CLB, ACTH, KD	*STXBP1*; AD	Developmental and epileptic encephalopathy 4 (612164)	No reduction in seizures
16	F	1 y 3 m	3 y 5 m	S, AA, F	Severe DD	VGB, VPA, TPM, ACTH, CLB	*GRIN2D;* AD	Developmental and epileptic encephalopathy 46 (617162)	No reduction in seizures
17	M	1 y 2 m	3 y 2 m	S, AA, M, T /LGS	Severe DD	VPA, ZNS, LTG, TPM, LEV, VGB, ACTH, PER, CLB, KD	*RANBP2;* AD	Encephalopathy, acute, infection-induced, 3, susceptibility to (608033)	No reduction in seizures
18	M	4 m	5 y 1 m	S, F	Severe DD	VGB, LTG, VPA, OXC, TPM, LEV, ACTH, KD	*TSC2*; AD	Tuberous sclerosis-2 (613254)	No reduction in seizures
19	F	1 y 3 m	3 y 3 m	AA, M, T, F, /LGS	Severe DD	LEV, LCM, VPA, OXC, TPM	*SZT2;* AR	Developmental and epileptic encephalopathy 18 (615476)	No reduction in seizures
20	M	6 m	4 y 5 m	F, AA, M, T, S/LGS	Severe DD	OXC, VPA, LTG, TPM, CZP, CLB, RFM, LCM	*CHRNA4;* AD	Epilepsy, nocturnal frontal lobe, 1 (600513)	No reduction in seizures

T, Tonic seizures; S, Spasms; F, Focal seizures; AA, Atypical absence; M, Myoclonic seizures; A, atonic seizures; DD, development dalay; ID, intellectual disability.

VPA, Valproic acid; TPM, Topiramate; CZP, Clonazepam; CLB, Clobazam; RFM, Rufinamide; LCM, Lacosamide; VGB, Vigabatrin; LTG, Lamotrigine; PER, Perampanel; ACTH, Adrenocorticotropic hormone; KD, Ketogenic diet. CBZ, Carbamazepine; PB, Phenobarbital; LEV, Levetiracetam; ZNS, Zonisamide; OXC, Oxcarbazepine; GTCS, General tonic-clonic seizures; LGS, Lennox-Gastaut Syndrome; DS, Dravet syndrome; PN, Patient number; y, year; m, month. AD, autosomal dominant. AR, autosomal recessive. XLD, X-linked dominant.

### Seizure outcomes after VNS treatment

**Figure 1:**
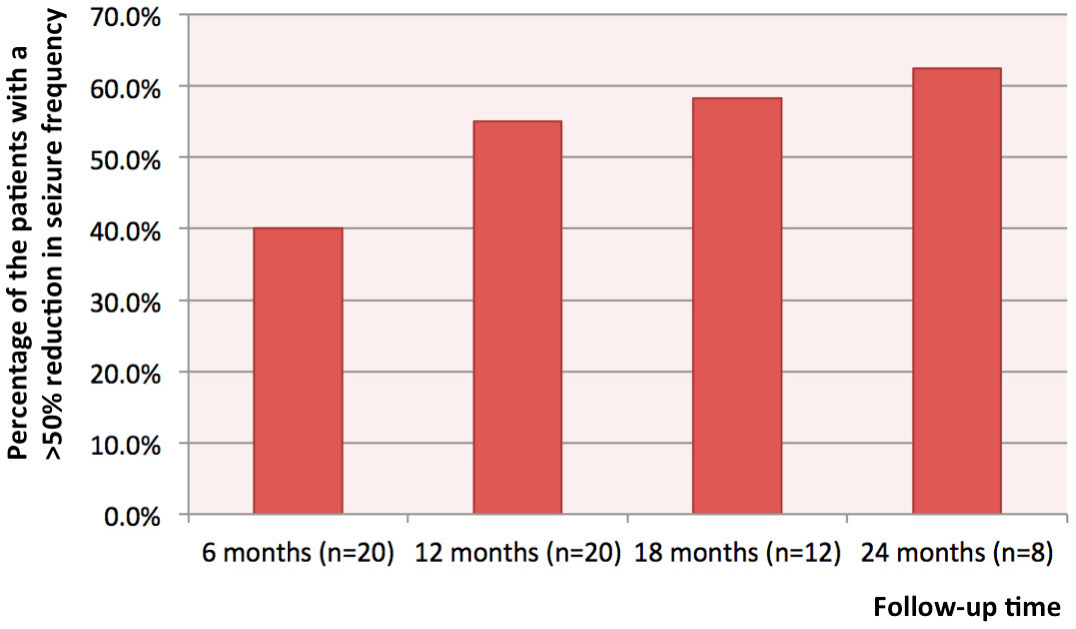
The percentages of the patients with a >50% reduction in seizure frequency at 6, 12, 18 and 24 months after stimulator implantation.

The median follow-up time was 1.4 years (range: 1.0–2.9). At 6, 12, 18, and 24 months after stimulator implantation, the responder rate of VNS was respectively 40.0% (8/20), 55% (11/20), 58.3% (7/12), 62.5% (5/8) (Figure 1). Two patients (10%, 2/20), with one carrying a pathogenic *SLC35A2* variant and the other a duplicated *MECP2*, were seizure-free at 12 months after stimulator implantation. Before stimulator implantation, the seizure frequency of the patient with a *SLC35A2* variant was 3–4 times per day, and the seizure frequency of the one with a duplicated *MECP2* was 1–2 times per day. For the eight patients with 24-month follow-up, the rate of seizure freedom was 12.5% (1/8). Among patients with DS caused by an *SCN1A* variant, 75.0% (3/4) had a >50% reduction in seizure frequency; and among those with a *DNM1* variant, 50.0% (1/2) had a >50% decrease in seizure frequency. There was only one patient each with the *CIC, MBD5, TUBGCP6, EEF1A2*, and *CHD2* gene variants, and all were VNS responders. Only one patient each harbored *STXBP1, LGI1, GRIN2D, RANBP2, TSC2, SZT2*, and *CHRNA4* variants, and none were responders.

At 6 months after stimulator implantation, the response rate of generalized seizures was 32.5%, and the rate of focal seizures was 37.5%. At 12 months after stimulator implantation, the response rate of generalized seizures was 47.5%, and the rate of focal seizures was 50.0%. The response rates of spasms, myoclonic, tonic, generalized tonic-clonic seizure (GTCS), atypical absence and atonic seizures were shown in Table 2.

**Table 2:** The response rates of each seizure type.

	**Response rate (6 months after VNS)**	**Response rate (12 months after VNS)**
Focal seizure	35.7% (5/14)	50.0% (7/14)
GTCS	50.0% (2/4)	75.0% (3/4)
Tonic seizure	28.6% (2/7)	42.9% (3/7)
Spasms	33.3% (3/9)	33.3% (3/9)
Myoclonic seizure	25.0% (2/8)	50.0% (4/8)
Atypical absence	33.3% (3/9)	44.4% (4/9)
Atonic seizure	33.3% (1/3)	66.7% (2/3)

GTCS, generalized tonic-clonic seizure.

Of the 20 patients, three had status epilepticus (SE); two harbored *SCN1A* variants and the other had a *DNM1* variant. Following VNS treatment, one patient with an *SCN1A* variant experienced prolongation of the interval between SE episodes from once a month to once every 3–4 months. The other two patients had no change in frequency of SE.

### Improvement of EEG following VNS

**Figure 2:**
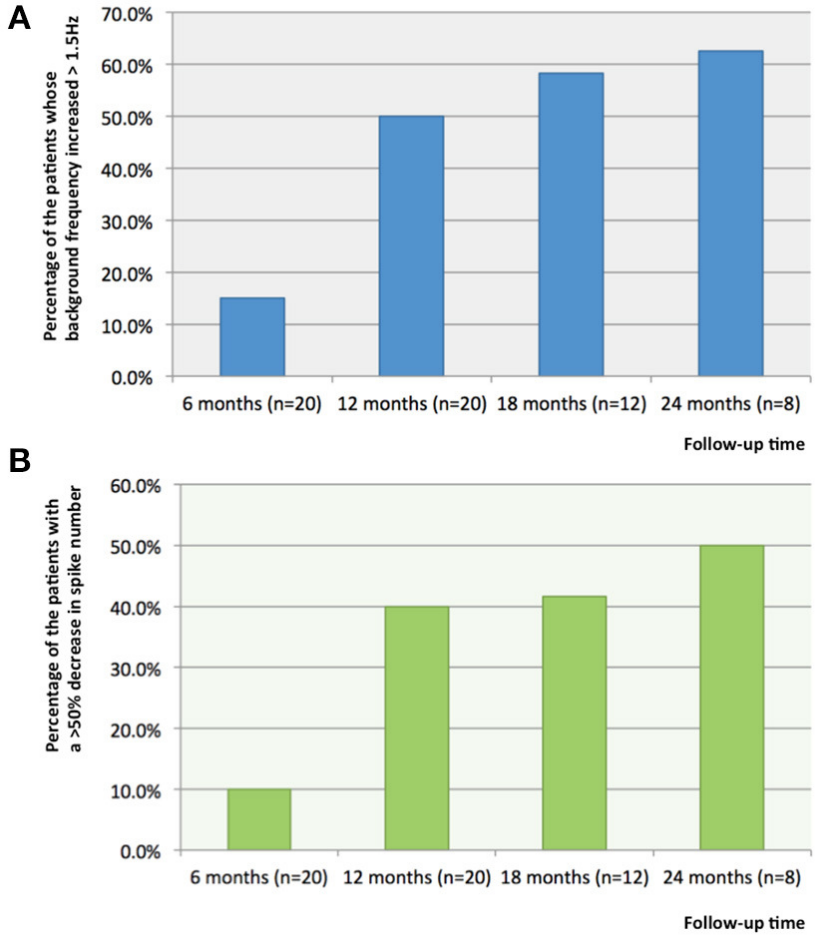
The percentages of the patients with increased background frequency and decreased number of spikes on interictal EEG. **(A)** Compared with the baseline data of patients before stimulator implantation, at 6, 12, 18, and 24 months after stimulator implantation, the percentage of the patients whose background frequency increased >1.5Hz was respectively, 15.0, 50.0, 58.3 and 62.5%; and **(B)** Compared with the baseline data, at 6, 12, 18, and 24 months after stimulator implantation, the percentage of the patients whose interictal EEG showed a >50% decrease in spike number was respectively, 10, 40.0, 41.6 and 50.0%.

Compared with the baseline data of patients before stimulator implantation, at 6, 12, 18, and 24 months after stimulator implantation, the percentage of the patients whose background frequency increased >1.5 Hz was respectively 15.0% (3/20), 50.0% (10/20), 58.3% (7/12) and 62.5% (5/8) (Figure 2A). Compared with baseline EEG, the background frequency improved significantly after 12 months of VNS treatment (*p* = 0.026). All the patients whose background frequency was improved were responders. The analysis of baseline EEG showed that the median frequency of background frequency was higher in the responders (responders 6.5 Hz and non-responders 4.5 Hz). Compared with the baseline data, at 6, 12, 18, and 24 months after stimulator implantation, the percentage of the patients whose interictal EEG showed a >50% decrease in spike number was 10% (2/20), 40.0% (8/20), 41.6% (5/12) and 50.0% (4/8) (Figure 2B). There was no statistical difference of spike number among various time points (*p* = 0.079). All the patients whose spike number decreased were responders. For the nine patients with no response to VNS treatment, there was no difference in terms of spike number and background frequency between preoperative and postoperative EEG (*p* > 0.05).

### Developmental outcomes following VNS treatment

Prior to stimulator implantation, we used the Griffiths Mental Development Scales or Wechsler Intelligence Scales to evaluate mental and motor development in the patients. For the 20 patients, we re-evaluated their development at 6 and 12 months after stimulator implantation. None of them obtained improvement according to the scales. However, compared to the developmental level at the preoperative evaluation, we found 5/20 patients (25%) reached new milestones or acquired new skills after VNS treatment; all five patients were responders and achieved developmental progression as seizure frequency decreased. These patients harbored *MECP2, SLC35A2, CIC, MBD5*, or *TUBGCP6* variants. The nine patients with no response to VNS did not acquire any new skills.

### Adverse effects of VNS treatment

In the early stage of VNS treatment, a few patients had adverse effects, but as the treatment continued, the adverse effects would be alleviated. At 6 months after stimulator implantation, 20.0% (4/20) of the patients had adverse effects including hoarseness (*n* = 3) and cough (*n* = 1). At 12 months after stimulator implantation, only 15.0% (3/20) of the patients had mild hoarseness.

### VNS parameter

At 12 months after stimulator implantation, 11 patients (55.0%) were responders. In the 11 responders, the median pulse amplitude was 2.15 mA (range: 1.0–2.7), the pulse width was usually 250/500 μs, and the pulse frequency was 30 Hz. The median duty cycle of the responders was 13% (range: 10–24%). In the patients with no response to VNS, the median pulse amplitude was 2.0 mA (range: 1.5–2.5), and the median duty cycle of the responders was 24% (range: 10–37%). Other parameters of the patients with no response were usually 250/500 μs and 30 Hz.

## Discussion

### VNS response rate in monogenic etiologies of DRE

In our study of 20 children with DRE of monogenic etiology, the responder rate to VNS at 12 months after stimulator implantation was 55.0% and the seizure-free rate was 10.0%, comparable with the overall efficacy of VNS in pediatric patients with DRE. A meta-analysis of 101 studies on VNS and seizure outcomes in pediatric DRE reported a response rate and seizure-free rate at last follow-up (mean: 2.54 years) of 56.4 % (95% confidence interval [CI]: 52.4–60.4) and 11.6% (95% CI: 9.6–13.9), respectively (6). In a VNS quality registry study of 436 epilepsy patients (52.5% adults and 47.2% children), the responder rate was 60% for the whole sample and 47% for patients with a genetic etiology (7), which was similar to the responder rate in our cohort. Although previous studies have shown that VNS may be more effective in patients with DRE of structural rather than unknown etiology (8, 9), there is no evidence to date that patients with a genetic etiology may derive greater benefit from VNS treatment.

Previous studies have shown that VNS is effective in both generalized and focal seizures (10). A meta-analysis of the VNS efficacy of 3,321 adults and children with DRE has showed that VNS is better for generalized seizures (8). In our study, we have found that there are no different VNS efficacies between generalized and focal seizures (*p* > 0.05). For generalized seizures, we have found that VNS might be better for GTCS and atonic seizures (Table 2), though there is no statistical difference of VNS efficacy among different seizure types (*p* > 0.05).

### VNS efficacy in patients with Dravet syndrome (DS) or tuberous sclerosis complex (TSC)

Patients with Dravet syndrome (DS) or tuberous sclerosis complex (TSC) account for the greatest proportion of patients with DRE of monogenic etiology who are referred for VNS treatment. A meta-analysis has evaluated the efficacy of VNS in this population. A meta-analysis of 216 patients including 92 with DS and 63 with TSC showed that 41% of the DS group responded to VNS treatment (11). Our study included four patients with DS; among them, three (75%) were responders. These findings suggest that VNS is to some extent effective for patients with DS, and should be considered as a treatment option. The meta-analysis also showed that the response rate among TSC patients was 68%; in one study of 20 patients with TSC, 40% achieved seizure freedom (11). These results suggest that VNS is a potential treatment option for TSC patients who cannot undergo or have a poor outcome after resection.

### Efficacy of VNS in DRE of other monogenic etiologies

We identified patients with DRE of other monogenic etiologies. The efficacy of VNS in some cases of *CDKL5-* or *MECP2*-related epilepsy, Angelman syndrome, and Ring chromosome 20 syndrome or in DRE of other monogenic etiologies (one case) have been previously reported (12–23).

*CDKL5* deficiency disorder is caused by *CDKL5* variants and is characterized by drug-resistant seizures and global developmental delay. A study of VNS efficacy in patients with a *CDKL5* variant showed that 69% (25/36) achieved symptom improvement following VNS treatment including a decrease in seizure duration (72%, 18/25) and seizure frequency (68%, 17/25), but the degree of reduction in seizure frequency was not specifically described in the study (12). A case report described an 8-year-old girl with a *CDKL5* variant who had an apparent reduction in seizure frequency and epileptic discharges with VNS treatment (13). However, a study of anti-seizure treatment in 29 patients with a *CDKL5* variant showed that of three patients treated by VNS, none had a reduction in seizure frequency (14). Therefore, the efficacy of VNS in patients with a *CDKL5* variant remains to be determined.

*MECP2*-related diseases include Rett syndrome and *MECP2* duplication syndrome. Rett syndrome is a genetic disorder characterized by profound cognitive impairment, communication deficits, stereotypical hand movements, gait abnormalities, and reduced head growth after a period of normal development for the first 6 to 18 months of life; it is primarily caused by *MECP2* variants on Xq28, and commonly affects females (15). A retrospective study on the efficacy of VNS in seven children with Rett syndrome with a median age of seizure onset of 34 months (range: 2–64 months) and median age at stimulator implantation of 9 years (range: 1–14 years) found that at the 12-month follow-up, 86% of patients had a reduction in seizure frequency of ≥50% (15). A meta-analysis of 11 patients with Rett syndrome also showed that 82% of patients had a ≥50% reduction in seizure frequency (11). *MECP2* duplication syndrome is characterized by cognitive impairment, seizures, autism, sleep disturbances and lower-respiratory-tract infections. In our study, one male patient had a duplication of X q28 (*MECP2*) and was diagnosed as *MECP2* duplication syndrome. He achieved seizure-free status after VNS treatment. Thus, patients with DRE harboring a *MECP2* variant may have a higher rate of response to VNS. VNS may also be recommended to patients with a *MECP2* variant for seizure control.

Some patients with chromosomal abnormalities were treated by VNS. Angelman syndrome (AS) is a neurogenetic disease caused by methylation of or microdeletion at maternally inherited 15q11–q13 or *UBE3A* variants (16), and is characterized by epileptic seizures, developmental delay, happy demeanor, motor deficits, etc. A case report of VNS efficacy in AS showed that all three patients under study had a decrease in seizure frequency and one became seizure-free following VNS treatment (16). Another case with AS had a >50% reduction in seizures following VNS (17). However, in a study of 16 patients with AS, only 19% showed improvement after the treatment (18). Given these inconsistent results, additional studies with a large sample are needed to establish the efficacy of VNS in patients with AS. Ring chromosome 20 syndrome is a rare chromosomal disorder characterized by developmental delay and DRE; in one report, 50% of patients had a >50% reduction in seizures and 25% were seizure-free after VNS (19–22). As patients with ring chromosome 20 syndrome often have DRE, they may benefit from VNS treatment.

There is little information on the efficacy of VNS for DRE of other monogenic etiologies. There is one report of a patient harboring a *KCNT1* variant and diagnosed with malignant migrating partial seizures in infancy who had a >90% reduction in seizure frequency after stimulator implantation but showed no improvement in mental and motor development (23). *SLC35A2* is an X-linked gene that encodes the Golgi-localized UDP-galactose transporter; variants of this gene can cause a congenital glycosylation disorder (24). Most patients with *SLC35A2* variants are female; male patients usually have a more severe phenotype. Our patient was male and had various seizure types such as spasms, focal seizures, and atypical absence along with severe development delay. He was free of seizures after VNS treatment, suggesting that DRE caused by *SLC35A2* variants can benefit from VNS treatment. Given the rarity of etiologies, it is difficult to evaluate the efficacy of VNS in cases of DRE of rare monogenic etiology.

### Efficacy of VNS in a pair of monozygotic twins

A pair of monozygotic twins (both girls) had the same phenotype and gene variant (*DNM1*, c. 854T>G, p. Leu285Arg, *de novo*, autosomal dominant inheritance) but responded differently to VNS: one patient achieved a 60% reduction in seizure frequency but the other had no reduction. A possible explanation for this is that while the twins had the same genotype, they were differentially affected by intrauterine factors and nutrition. The finding that monozygotic twins respond differently to VNS complicates the identification of factors influencing treatment response and suggests that the efficacy of this procedure is not determined solely by genetic background, but is affected by many factors.

### Limitations

There were some limitations to this study. First, the number of cases with each pathogenic gene was small because of the rarity of the variants. Second, part of the clinical data was retrospective.

## Conclusions

In our cohort of 20 children with DRE of monogenic etiology, at 12 months of follow-up, the rate of response to VNS was 55% and the seizure-free rate was 10%, which is comparable to the overall efficacy of VNS in children with DRE. This is the first report demonstrating that patients with DRE harboring *SLC35A2, CIC, DNM1, MBD5, TUBGCP6, EEF1A2*, and *CHD2* variants may benefit from VNS treatment. VNS is also a treatment option for patients with DS, TSC, or Rett syndrome. However, the efficacy of VNS for DRE of other monogenic etiologies is uncertain because of the rarity of these variants. A prospective registry study with a large patient sample can provide more detailed insight into the efficacy of VNS for DRE of monogenic etiology.

## Data availability statement

The original contributions presented in the study are included in the article/supplementary material, further inquiries can be directed to the corresponding author/s.

## Ethics statement

The studies involving human participants were reviewed and approved by the Medical Ethics Committee of Peking University First Hospital. Written informed consent to participate in this study was provided by the participants' legal guardian/next of kin.

## Author contributions

YW and HX designed the study and wrote the manuscript. YW, HX, JM, and TJ performed parameter adjustment of VNS and collected the data. LC and QL conducted stimulator implantation. YW, HX, and JM analyzed the data. All authors contributed to the article and approved the submitted version.

## Funding

This work was supported by National Key Research and Development Program (2021YFC2401202).

## Conflict of interest

The authors declare that the research was conducted in the absence of any commercial or financial relationships that could be construed as a potential conflict of interest.

## Publisher's note

All claims expressed in this article are solely those of the authors and do not necessarily represent those of their affiliated organizations, or those of the publisher, the editors and the reviewers. Any product that may be evaluated in this article, or claim that may be made by its manufacturer, is not guaranteed or endorsed by the publisher.
